# Large-Scale Geographic Variation in Distribution and Abundance of Australian Deep-Water Kelp Forests

**DOI:** 10.1371/journal.pone.0118390

**Published:** 2015-02-18

**Authors:** Ezequiel M. Marzinelli, Stefan B. Williams, Russell C. Babcock, Neville S. Barrett, Craig R. Johnson, Alan Jordan, Gary A. Kendrick, Oscar R. Pizarro, Dan A. Smale, Peter D. Steinberg

**Affiliations:** 1 Sydney Institute of Marine Science, Mosman, New South Wales, Australia; 2 Centre for Marine Bio-Innovation and School of Biological, Earth and Environmental Sciences, University of New South Wales, Sydney, New South Wales, Australia; 3 Australian Centre for Field Robotics, University of Sydney, Sydney, New South Wales, Australia; 4 CSIRO Marine and Atmospheric Research, Brisbane, Queensland, Australia; 5 Institute for Marine and Antarctic Studies, University of Tasmania, Hobart, Tasmania, Australia; 6 Department of Primary Industries, New South Wales Government, Port Stephens Fisheries Institute, Nelson Bay, New South Wales, Australia; 7 Oceans Institute and School of Plant Biology, University of Western Australia, Perth, Western Australia, Australia; 8 Marine Biological Association of the United Kingdom, The Laboratory, Citadel Hill, Plymouth, United Kingdom; University of Waikato (National Institute of Water and Atmospheric Research), NEW ZEALAND

## Abstract

Despite the significance of marine habitat-forming organisms, little is known about their large-scale distribution and abundance in deeper waters, where they are difficult to access. Such information is necessary to develop sound conservation and management strategies. Kelps are main habitat-formers in temperate reefs worldwide; however, these habitats are highly sensitive to environmental change. The kelp *Ecklonia radiate* is the major habitat-forming organism on subtidal reefs in temperate Australia. Here, we provide large-scale ecological data encompassing the latitudinal distribution along the continent of these kelp forests, which is a necessary first step towards quantitative inferences about the effects of climatic change and other stressors on these valuable habitats. We used the Autonomous Underwater Vehicle (AUV) facility of Australia’s Integrated Marine Observing System (IMOS) to survey 157,000 m^2^ of seabed, of which *ca* 13,000 m^2^ were used to quantify kelp covers at multiple spatial scales (10–100 m to 100–1,000 km) and depths (15–60 m) across several regions *ca* 2–6° latitude apart along the East and West coast of Australia. We investigated the large-scale geographic variation in distribution and abundance of deep-water kelp (>15 m depth) and their relationships with physical variables. Kelp cover generally increased with latitude despite great variability at smaller spatial scales. Maximum depth of kelp occurrence was 40–50 m. Kelp latitudinal distribution along the continent was most strongly related to water temperature and substratum availability. This extensive survey data, coupled with ongoing AUV missions, will allow for the detection of long-term shifts in the distribution and abundance of habitat-forming kelp and the organisms they support on a continental scale, and provide information necessary for successful implementation and management of conservation reserves.

## Introduction

Most of the world’s ecosystems are dominated by habitat-forming species that facilitate other organisms by modifying the surrounding environment [1]. These organisms, such as trees on land and reef-building corals and large perennial seaweeds in the oceans, support extremely diverse and abundant communities, which are among the most productive on Earth [2–4]. Multiple stressors are negatively affecting these habitat-forming species in many systems, leading, in turn, to declines in local biodiversity and the related ecological goods and services [5–8]. Ecological information on habitat-formers and the processes influencing them is therefore crucial for successful management and conservation.

Stressors vary, however, with respect to the spatio-temporal scale at which they can impact natural systems [9]. Large-scale spatial and temporal studies are therefore needed to understand how stressors such as climatic change affect major habitat-formers and the biodiversity they support. On land, the development of remote sensing technologies has enabled description of large-scale ecological patterns of key habitat-formers, *e.g*. trees, in different environments [10]. These patterns are now the baseline against which changes related to the climate and other stressors are measured [2]. In subtidal marine systems, studies at such scales are rare, but much needed. Most studies of effects of climatic change on organisms involve small-scale surveys and/or manipulative experiments, which are difficult to conduct at larger scales [11]. This is potentially problematic, because processes important for structuring assemblages in small-scale studies may not be generalisable to larger scales due to the intrinsic spatial and temporal variability of natural systems [12]. Thus, while small-scale manipulative experiments are necessary to establish cause and effect, they need to be coupled with large-scale surveys to allow understanding of large-scale effects of climatic changes and other large-scale disturbances, such as fishing and other resource exploitations, changes resulting from land-use practices, etc. [13–15].

In marine subtidal systems, descriptions of large-scale patterns are limited by available technologies. Surveys for testing hypotheses focused on patterns or processes manifesting at large scales are typically done *via* SCUBA diving [16, 17]. However, the spatial coverage of such surveys either at the local sampling scale or the geographic spread is often insufficient to examine large-scale processes (but see [18]), particularly for communities deeper than 30 m which are difficult to access to any great extent *via* SCUBA diving.

Recently, autonomous underwater vehicles (AUVs) have been used successfully to quantify patterns of benthic biodiversity at large spatial scales with great spatial accuracy (<1 m), over large geographical and/or in deep areas largely inaccessible by SCUBA diving [19, 20]. In Australia, the Integrated Marine Observing System (IMOS) supports an AUV facility with the objective of assessing the effects of climatic change on benthic assemblages at reference stations around the continent, encompassing tropical and temperate reefs [21].

Subtidal temperate reefs are generally dominated by habitat-forming kelps and the wide variety of organisms these support [6, 8]. Kelp forests are among the most diverse and productive coastal habitats [6, 22, 23], but they are declining along several coastlines across the globe due to stressors such as eutrophication, overfishing and ocean warming [6, 24–26].

The kelp *Ecklonia radiata* (hereafter ‘*Ecklonia*’) is a major habitat-former that characterizes subtidal rocky reefs in subtropical and temperate Australia [27]. At local scales, kelp declines are influenced by different stressors, such as eutrophication on the south coast and herbivory on the east coast [24, 28, 29]. At a larger scale, ocean warming is a major stressor on both coasts, and it is likely to interact with these and other stressors acting at smaller scales [24, 26].

Most kelp studies have, however, focused on shallow-water populations (usually <15 m) due to the difficulties in accessing deeper-water habitats (see above). The abundance and distribution of *Ecklonia* in deeper waters and the extent to which these populations are affected by stressors such as climatic change are poorly understood. The strengthening of the major boundary currents (Leeuwin Current and Eastern Australian Current) is leading to increased water temperatures and decreased nutrient levels in both coasts [24, 26]. Such changes may result in shifts in kelp distribution southward and/or into deeper waters, driven by their physiological requirements and ecological processes such as recruitment, which can be negatively influenced by high water temperatures, and herbivory, which could potentially increase as a result of range expansion of tropical herbivores [26, 30, 31].

The aim of this study was to establish, for the first time, patterns of abundance and distribution of kelp forests in deeper waters (>15 m—<80 m) at a large scale, and provide baseline ecological data necessary to make quantitative inferences about the long-term effects of climatic change and other stressors on kelp and the organisms it supports. We surveyed rocky reefs and quantified kelp cover at several spatial scales and depths along the West and East coast of Australia using the IMOS AUV, encompassing almost the entire latitudinal range of this species in the continent. In addition, we examined relationships between kelp cover and relevant physical variables to determine potential abiotic drivers. Finally, we conducted targeted surveys of kelp forests in the northern limit of their distribution in the East coast to quantify kelp abundance and its variability, as populations in the northern limit of the distribution are likely to be more susceptible to warming.

## Materials and Methods

The AUV *Sirius* (Australian Centre for Field Robotics, University of Sydney) was used to survey rocky reefs across temperate Australia. This AUV, which is supported by IMOS, is 2 m long x 1.5 m height, weighs 250 kg and maintains an altitude of ∼2 m above the substratum at ∼0.5 m/s while photographing the benthos approximately every second (detailed information in [21]). During these surveys, geo-referenced images were taken at an average of 2.1 m above the substratum, each image covering an area of 2.2 m^2^ with a spatial resolution of ∼1 mm/pixel. In addition, the AUV carries an onboard Seabird 37-serial interface (SI) conductivity and temperature sensor, and a Wetlabs Ecopuck fluorometer-turbidity sensor to measure chlorophyll-*a* and coloured dissolved organic matter (CDOM). All data obtained by the AUV is freely available at the IMOS Ocean Portal (*http://imos.aodn.org.au*).

### Regions surveyed

**Fig 1:**
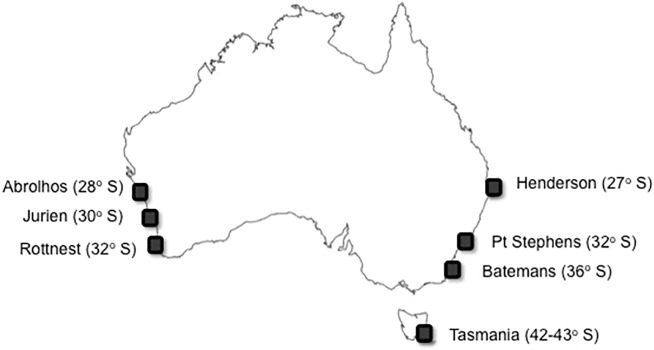
Regions sampled. Map of Australia showing the regions on the West (Abrolhos, Jurien, Rottnest) and the East coast (Henderson, Port Stephens, Batemans, Tasmania) surveyed by the AUV in 2010.

Surveys of Australian subtropical and temperate rocky reefs were done at three regions separated by 2° latitude along the West coast: Abrolhos Islands (‘Ab’; 28° S), Jurien Bay (‘Ju’; 30° S), and off Rottnest Island (‘Ro’; 32° S) in Western Australia (WA), and at four regions separated by 4–6° latitude along the East coast: Henderson (‘He’; 27° S) in Queensland (Qld), Port Stephens (PS; 32° S) and Batemans Bay (Ba; 36° S) in New South Wales (NSW), and Tasmania (Ta; 42–43° S; Fig. 1), thus encompassing almost the entire kelp latitudinal range along both coasts of the continent. Populations in the south of the continent were not surveyed because our main interest relied on quantifying latitudinal variation as climatic change is likely to mainly affect latitudinal, rather than longitudinal variation in kelp distribution and abundance. Given the non-intrusive nature of the research and that AUV surveys were undertaken in Australian state waters, many jurisdictions did not require a permit. Permits for specific jurisdictions were: 95170, Qld Department of Primary Industries and Fisheries for CSIRO Marine and Atmospheric Research; P00/0054–6.0, NSW Department of Primary Industries (Fishing and Aquaculture) to The University of New South Wales, CMB; A12514, University of Tasmania animal ethics committee for NERP Marine Biodiversity Hub research. The study did not involve endangered or protected species. All sites were coastal, with the exception of the Abrolhos Islands (northern WA, ∼60 km from the coast), and distances from the shore typically ranged between 500 m to 10 km depending on the slope of the reefs. GPS coordinates of all locations surveyed are detailed in Table A in S1 Appendix.

All surveys were conducted in 2010, but the timing of each survey varied among regions: all three regions on the West coast were surveyed in April; Tasmania was surveyed in June; Henderson, Port Stephens and Batemans were surveyed between late October-early December. Although kelp distribution is unlikely to vary seasonally which thus allows formal comparisons among regions along the continent, *Ecklonia* growth and biomass do vary seasonally in a pattern that seems consistent in both coasts and may thus influence cover estimates [32–34]. Therefore, formal comparisons of kelp cover or physical variables measured *in situ* between regions sampled in different seasons were not attempted because these would be temporally confounded. Additionally, no formal comparisons were made between Henderson and other regions because areas surveyed in this region were specifically chosen for the presence of kelp (see below).

In each region, kelp cover and physico-chemical variables were measured at a constant depth of ∼30 m within replicate areas of 25 x 25 m (grids). In addition, these variables were measured along the depth gradient using transects from ∼15 to up to 80 m depth (East coast) or using grids at 15, 30 and 40–45 m (West coast). Surveys in grids or transects are explained in detail below.

### Grids at 30 m depth

Two rocky reef locations ∼3–20 km apart were randomly selected within each region (except for Tasmania, with five locations ∼20–80 km apart) and 2–3 25 x 25 m grids separated by 50–200 m were surveyed within each location at a depth of ∼30 m. The AUV provided full coverage of the seabed within each 25 x 25 m area. A random subset of 100 images per grid was selected (except for Tasmania, where *n* = 20 as determined sufficient to reliably estimate kelp cover from pilot studies in this region) and kelp cover was quantified using 50 random points over each image.

The areas surveyed were chosen based on the presence of rocky reef habitat (typically extensive bedrock) determined from swath acoustic surveys [35, 36], although this depended on the availability/precision of such data. Due to the patchy structure of reef habitat in some locations, some grids had high proportion of sand habitat, so only grids with >60% cover of reef habitat were used, resulting in a total of 61 grids analysed (Table A in S1 Appendix).

### Transects along the depth gradient

In addition to surveyed grids at 30 m, on the East coast, two transects (200–500 x 1.5 m; 0.5–5 km apart) were surveyed in each region along the depth gradient to quantify kelp cover across depth. One in every 100^th^ image (*i.e*. one image every ∼150 m) was selected from each transect (PS: *n* = 32–36; Ba: *n* = 24–70; Ta: *n* = 67–105 images per transect) and the percentage cover of kelp and sand were quantified as described above. On the West coast, transects along the depth gradient were not feasible due to the low relief of the coastline and the gentle slope of the coastal shelf, and instead 2–3 25 x 25 m grids were surveyed as described above at two additional depths (15, 40–45 m) in each region. Of these, only grids with >60% cover of reef habitat were used for analyses (Table A in S1 Appendix).

### Approximate northern limit of kelp distribution on the East coast

To quantify kelp abundance and estimate variability at several spatial scales at the approximate northern limit of this species’ distribution, reefs purposely targeted for the presence of kelp were surveyed in Henderson, SE Qld. In this region, 3 25 x 25 m grids at ∼30 m depth were nested in each of two sites ∼500 m apart within each of two locations (∼5 km apart). Transects were also undertaken as described above (*n* = 16–58 images were analysed per transect).

### Physical variables

During the surveys, the AUV measured—essentially continuously—*in situ* temperature (°C), salinity (PSU), chlorophyll-*a* and coloured dissolved organic matter (CDOM) concentrations (mg/m^3^) using the sensors described above. These variables are deemed to be key for kelp survival and growth, with temperature and salinity directly influencing their physiology, and chlorophyll-*a* and CDOM used as a proxy for nutrients and light availability. One hundred *in situ* measurements for each variable were randomly selected from each grid and used for analyses. For transects, analyses were done on averages calculated for each depth-change of 1 m. Because *in situ* AUV measurements were not replicated in time and are likely to vary significantly at multiple temporal scales, independent measurements of sea-surface monthly averages of temperature (°C), chlorophyll-*a* concentration (mg/m^3^), CDOM index (unitless) and photosynthetically available radiation (PAR; Einstein/m^2^/day) quantified *via* remote sensing (Modis-Aqua 4 km) were also obtained from the Giovanni Ocean Colour Radiometry portal, National Aeronautics and Space Administration (NASA) to provide more representative estimates of these variables. Two locations separated by 0.1° latitude but within the longitudinal range of the areas surveyed by the AUV (Table A in S1 Appendix) were randomly chosen within each of the seven regions around Australia. For each location, we obtained the monthly average of each physical variable for each of the three summer (January-March) and winter (July-September) months across eight years (2003–2010). Thus, for each year, we had 3 summer and 3 winter values.

### Analyses of data

Analyses of variance (ANOVA) were used to examine differences in percentage cover of kelp or the magnitudes of physical variables (temperature, salinity, chlorophyll-*a*, CDOM) at ∼30 m depth among regions along the West and East coast, as well as among the three depth-ranges sampled on the West coast. ANOVA was also used to compare physical variables obtained through remote sensing among regions on the West and East coasts and between seasons. Analyses are explained in detail in each Table as designs varied depending on the variables analysed and the regions. Briefly, grids were nested in sites or locations, which were nested within regions, and all these factors were random except for region, which was fixed. Analyses in WA involved depth (15 *vs* 30 *vs* 40–45 m), which was a fixed factor. For analyses on remotely sensed data, seasons was fixed (summer *vs* winter) and years (2003–2010) was random. Because some of the sampled grids in some locations were mostly sand and thus excluded from analyses (see above), the design became unbalanced, which can increase the probability of type I error among other issues [37]. To avoid this, equal numbers of grids or locations were selected randomly from all those available in each region to make the analyses balanced. When Cochran’s test (*C*) for heterogeneity of variances was significant and no transformation was possible, ANOVA were still employed as it is robust to departures from the assumptions in balanced designs with large sample size and number of treatments [37]. Non-significant interactions with *P* > 0.25 were eliminated or pooled as appropriate [38]. Where significant interaction terms were detected, Student-Newman-Keuls (SNK) contrasts were used to determine which treatments differed [37]. Analyses were done using GMAV 5 (EICC, University of Sydney).

Pearson’s correlation was used to determine relationships among physical variables measured across depth for each region on the East coast. Simple and multiple linear regression analyses were used to determine relationships between kelp cover and all physical variables measured across depth at each of the four regions surveyed on the East coast. Regression analyses were not done for the West coast as depth was a categorical variable (15, 30, 40 m) and ANOVAs were done instead (see above). Marginal tests (simple linear regressions) were done to determine relationships between kelp cover and each physical variable independently of the others. *P*-values were calculated using 9,999 permutations. The proportion of the variability explained by all variables combined was obtained in sequential tests using R^2^ selection criterion and the Forward selection procedure. The overall best solution (*i.e*. the most parsimonious model) was obtained using the Bayesian Information Criterion (BIC), using all possible combinations of variables. Variables that were strongly inter-correlated (|r|>0.95) were excluded from the model, leaving one of these to represent the mutually correlated set. Regression analyses were done on Euclidean distance matrices constructed for each data, using DISTLM in the PERMANOVA add-on for PRIMER v6 [39].

## Results

Overall, ∼157,225 m^2^ of subtidal rocky reefs were surveyed along the West and East coast of sub-tropical and temperate Australia. Percentage covers of kelp *Ecklonia* were quantified using a subset of 6,094 AUV images, corresponding to a total of ∼13,410 m^2^.

### Kelp cover at 30 m and physical variables

**Fig 2:**
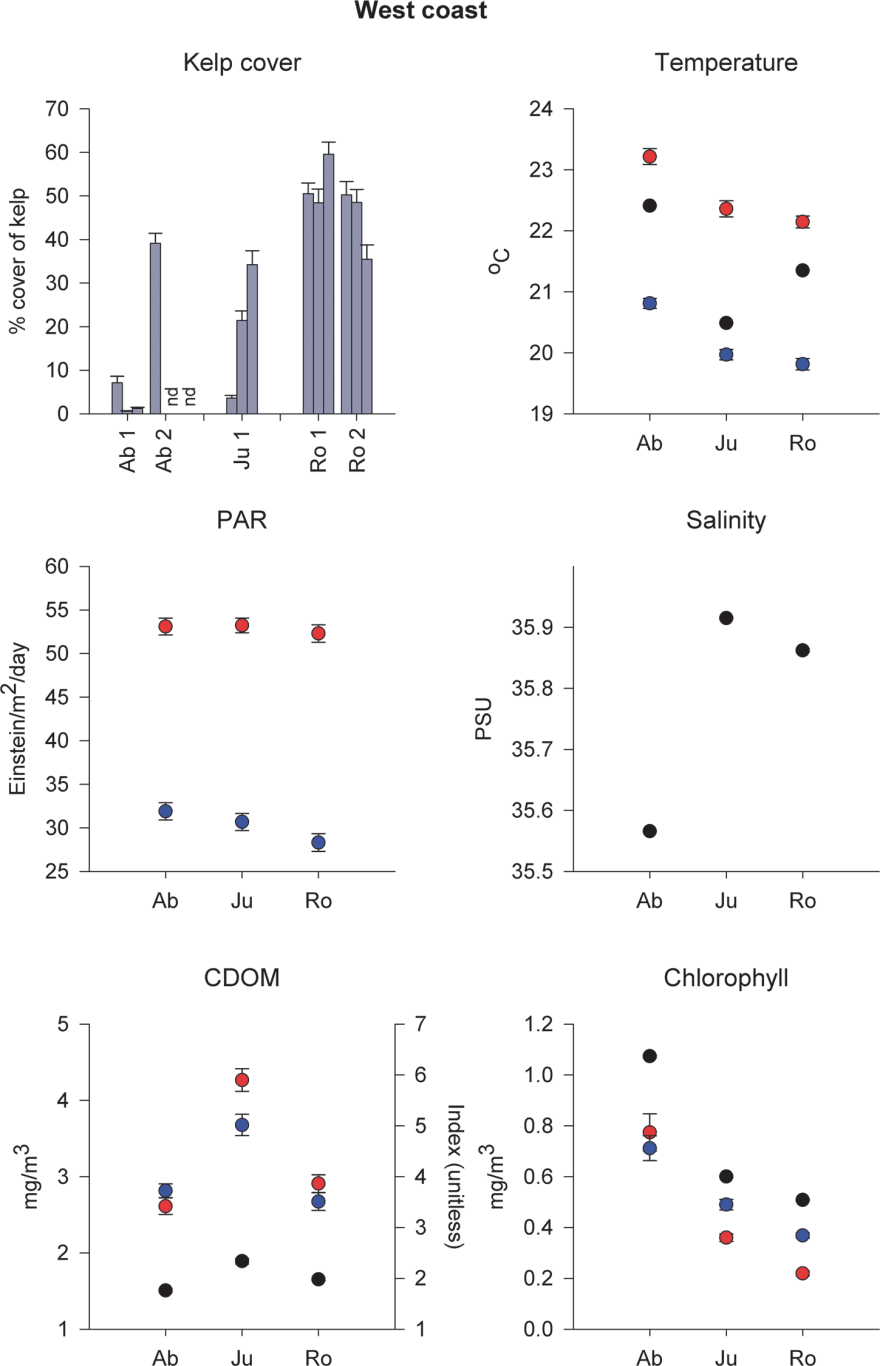
Kelp cover at 30 m depth and physical variables in West Australia. Percentage cover of kelp (mean + SE, *n* = 100) at 30 m depth for each grid at 1–2 locations in Abrolhos (Ab, 28° S), Jurien (Ju, 30° S) and Rottnest (Ro, 32° S), and physical variables measured *in situ* (temperature, salinity, chlorophyll and CDOM concentration; black symbols; mean ± SE, *n* = 300) or obtained from Modis-Aqua 4 km (NASA) sea-surface monthly averaged data (temperature, photosynthetically available radiation (PAR), chlorophyll and CDOM index) for summer (red symbols) and winter (blue symbols; mean ± SE, *n* = 48). nd, no data.

**Fig 3:**
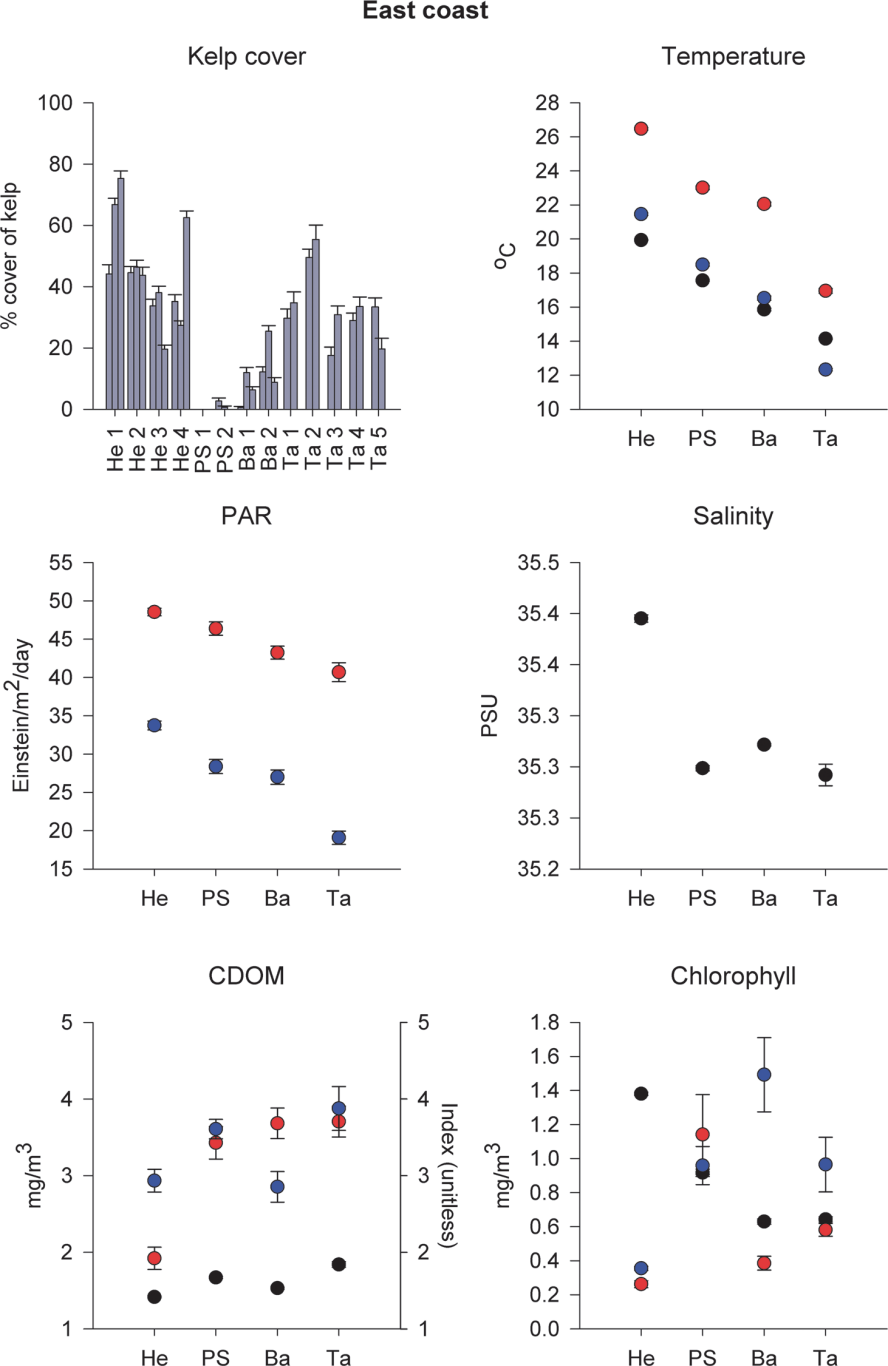
Kelp cover at 30 m depth and physical variables in East Australia. Percentage cover of kelp (mean + SE) at 30 m depth for each grid at 2–5 locations in Henderson (27° S), Port Stephens (PS, 32° S), Batemans Bay (Ba, 36° S; *n* = 100) and Tasmania (Ta, 42–43° S; *n* = 20), and physical variables measured *in situ* (temperature, salinity, chlorophyll and CDOM concentration; black symbols; mean ± SE, *n* = 300) or obtained from Modis-Aqua 4 km (NASA) sea-surface monthly averaged data (temperature, photosynthetically available radiation (PAR), chlorophyll and CDOM index) for summer (red symbols) and winter (blue symbols; mean ± SE, *n* = 48). Henderson is the approximate northern limit of kelp distribution and reefs here were specifically targeted for the presence of kelp.

Percentage cover of kelp generally increased with increasing latitude (Figs. 2 and 3). On the West coast, cover of kelp at 30 m was significantly greater at Rottnest (32° S) than at Jurien (30° S) or Abrolhos (28° S), despite significant variability among grids (separated by ∼100 m; Table 1A, Fig. 2).

**Table 1:** ANOVA of kelp cover at 30 m depth among regions separated by 2° to 4° Latitude in (a) Western Australia and (b) New South Wales, and (c) among locations in Tasmania.^#^

**(a)** Source	*Df*	MS	*F*	*P*
Region	2	192971	20.4	**0.002**
Grids(Re)	6	9455	20.3	**<0.001**
Residual	891	467		
*C* = 0.25, *P* < 0.01; Contrasts: Ro > Ju = Ab				
**(b)** Source	*df*	MS	*F*	*P*
Region	1	32013	9.1	**0.009**
*Location(Re)	2	6480	*	*
Grids(Lo(Re))	8	2797	25.6	**<0.001**
Residual	1188	110		
*C* = 0.25, *P* < 0.01				
**(c)** Source	*df*	MS	*F*	*P*
Location	4	2207	4.9	0.060
Grids(Lo)	5	454	4.4	**0.001**
Residual	190	102		
*C* = 0.18, *P* > 0.05 (Arcsin transformation)				

^#^ Non-significant terms with *P* > 0.25 (*) were eliminated. (a) Region is fixed with 3 levels (Ab, Abrolhos 28° S; Ju, Jurien 30° S; Ro, Rottnest 32° S), Grids is random, nested in Region, with 3 levels. The replicates are the images (*n* = 100). (b) Region is fixed with 2 levels (PS, Port Stephens 32° S; Ba, Batemans 36° S), Location is random, nested in Region with 2 levels, Grids is random, nested in Location, with 3 levels. The replicates are the images (*n* = 100). (c) Location is random with 5 levels (all between ∼ 42–43° S), Grids is random, nested in Location, with 2 levels. The replicates are the images (*n* = 20).

Water temperature and chlorophyll-*a* concentration measured *in situ* (30 m) decreased significantly with increasing latitude. The opposite pattern was found for salinity, although the range was only ∼0.3 PSU. CDOM concentration was greater at Jurien than at Abrolhos or Rottnest (Table B in S1 Appendix, Fig. 2). A similar pattern was observed for data obtained through remote sensing (Table C in S1 Appendix, Fig. 2). Differences in sea-surface temperature across regions were consistent in summer and winter. Satellite-derived chlorophyll-*a* concentration was greater in winter than in summer at Jurien and Rottnest, but not at Abrolhos, while CDOM only differed between seasons at Jurien, where it was greater in summer, although this varied across years (Table C in S1 Appendix). Finally, surface PAR did not differ among regions, but was significantly greater in summer than winter (Table C in S1 Appendix, Fig. 2).

On the East coast, kelp cover also increased with increasing latitude. Kelp cover at Batemans (36° S) was significantly greater than at Port Stephens (32° S; Table 1B), but the greatest cover was recorded in Tasmania (42–43° S; Fig. 3). Formal comparisons of kelp cover and, in particular, *in situ* physical variables between Tasmania and Batemans or Port Stephens were, however, not done because these would be temporally confounded (see Methods). Nevertheless, *Ecklonia* biomass is typically at its minimum in winter, suggesting estimates of greater cover in Tasmania (sampled in June) are conservative. Kelp cover varied significantly within these three regions at the spatial scale of grids (separated by ∼100 m), not locations (separated by kilometres; Table 1B,C).

There were no significant differences in *in situ* (30 m) temperature, salinity, chlorophyll-*a* or CDOM concentration between Batemans and Port Stephens (Table B in S1 Appendix), although, on average, temperature was ∼2°C colder and chlorophyll-*a* concentration was 30% lower at Batemans (Fig. 3). For remote sensing data, sea-surface temperature and PAR decreased with increasing latitude and was significantly different across all regions sampled along the East coast, both in summer and winter (Table C in S1 Appendix). Remote-sensed CDOM only differed among regions in winter, when it was generally lower in Batemans than in Port Stephens or Tasmania, and between seasons in Batemans, where it was greatest in summer (Table C in S1 Appendix), while chlorophyll-*a* differed among regions in summer, when it was generally higher in Port Stephens, and between seasons in Batemans, where it was lowest in summer (Table C in S1 Appendix, Fig. 3). Differences in remote-sensed CDOM and chlorophyll-*a* between regions and seasons varied, however, across years (Table C in S1 Appendix).

### Relationships between kelp cover and physical variables across depths

**Fig 4:**
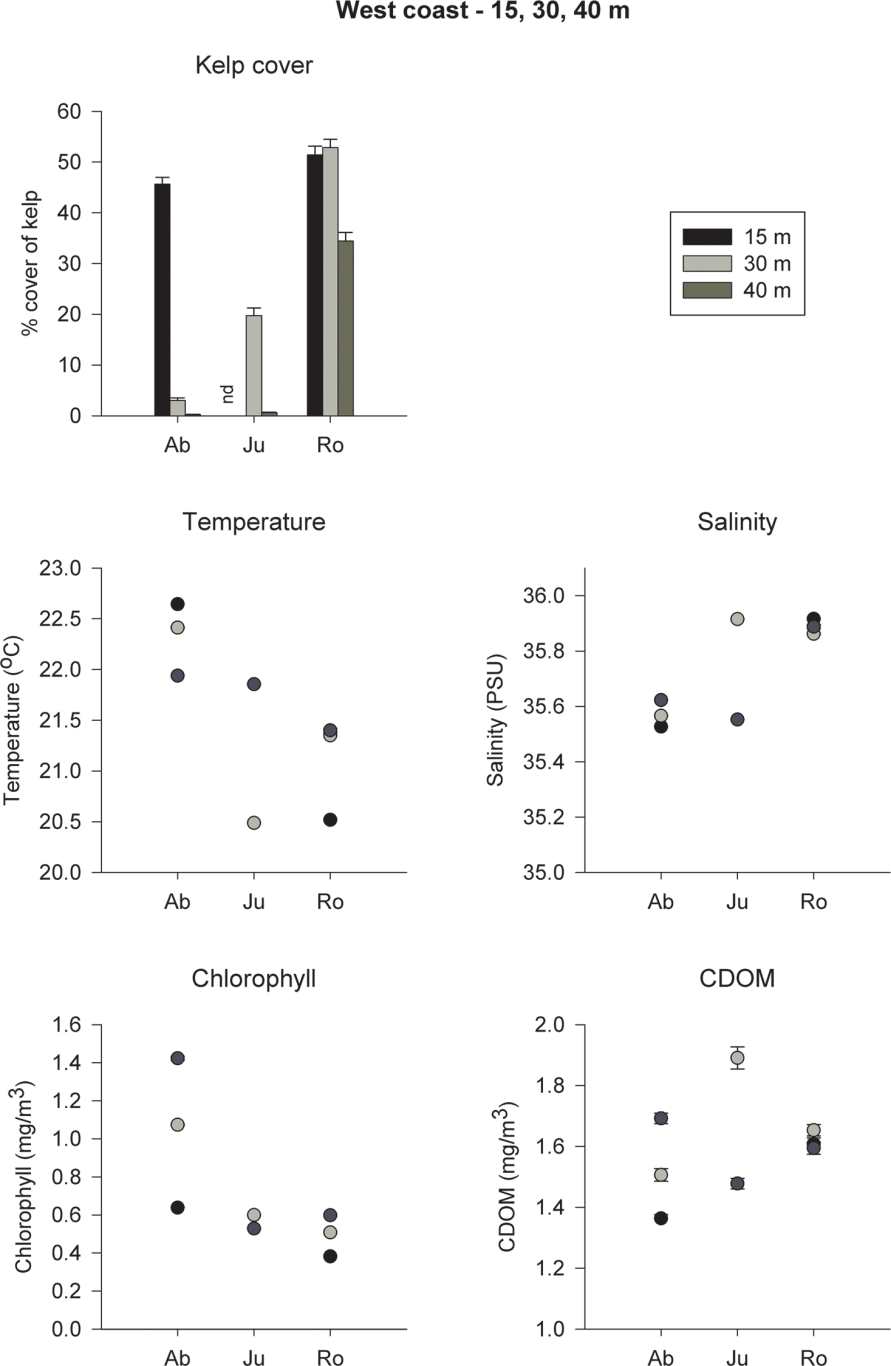
Kelp cover and physical variables across depth in West Australia. Mean (+ SE, *n* = 200–300) percentage cover of kelp, and *in situ* temperature, salinity, chlorophyll and CDOM concentration at 15 (black bars), 30 (grey bars) and 40 m (dark grey bars) depth in Abrolhos (Ab, 28° S), Jurien (Ju, 30° S) and Rottnest (Ro, 32° S) on the West coast of Australia. nd, no data.

On the West coast, kelp cover varied between regions and depths, although overall it was greater at Rottnest than at Jurien or Abrolhos, despite significant variability among grids (Table 2A,B, Fig. 4). Kelp cover at 30–40 m generally increased with latitude (Fig. 4). At Abrolhos, kelp cover was greater at 15 m than at 30 or 40 m. Kelp cover in Jurien was greater at 30 m than at 40 m. On the other hand, kelp cover at Rottnest did not differ among depths. On average, kelp cover at 15 m at Abrolhos was similar to that at Rottnest (Fig. 4).

**Table 2:** ANOVA of kelp cover at (a) 15, 30 and 40 m at Abrolhos and Rottnest and (b) at 30 and 40 m at Abrolhos, Jurien and Rottnest in Western Australia.^#^

	**(a)** 3 Depths, 2 Regions				**(b)** 2 Depths, 3 Regions			
Source	*df*	MS	*F*	*P*	*df*	MS	*F*	*P*
Region	1	372450	56	**<0.001**	2	277870	35	**<0.001**
Depth	2	138360	21	**<0.001**	1	74979	9	**0.015**
Re x De	2	74693	11	**0.004**	2	10973	1	0.312
Grids(Re x De)	11	6671	13	**<0.001**	11	7951	21	**<0.001**
Residual	1683	518			1683	378		
	*C* = 0.13, *P* < 0.01				*C* = 0.17, *P* < 0.01			
Contrasts:	Depth: Ab, 15 > 30 = 40; Ro, 15 = 30 = 40; Region: 15 m, Ab = Ro; 30 and 40 m, Ro > Ab				Depth: 30 > 40; Region: Ro > Ju = Ab			

^#^ (a) Region is fixed with 2 levels (Ab, Abrolhos 28° S; Ro, Rottnest 32° S), Depth is fixed, orthogonal, with 3 levels (15, 30, 40 m). Grids is random, nested in Region and Depth, with 3 levels except for Ab at 40 m, with 2 levels. The replicates are the images (*n* = 100). (b) Region is fixed with 3 levels (Ab, Abrolhos 28° S; Ju, Jurien 30° S; Ro, Rottnest 32° S), Depth is fixed, orthogonal, with 2 levels (30, 40 m). Grids is random, nested in Region and Depth, with 3 levels, except for Ab at 40 m, with 2 levels. The replicates are the images (*n* = 100).

Analyses for *in situ* temperature, salinity, chlorophyll-*a* and CDOM concentration also showed an interaction between regions and depth (Table D in S1 Appendix, Fig. 4). Temperature generally decreased with increasing latitude. At Abrolhos, temperature at 15 and 30 m were ∼1°C higher than at 40 m. At Rottnest, temperature at 15 m was ∼1°C lower than at deeper waters (30–40 m). Chlorophyll-*a* concentration was generally lowest at 15 m in both regions. In contrast, salinity generally increased with latitude, but did not differ among depths. Concentration of CDOM was greater at Rottnest than at Abrolhos only at 15 m; there were no differences among regions for other depths or among depths in each region (Table D in S1 Appendix, Fig. 4).

**Fig 5:**
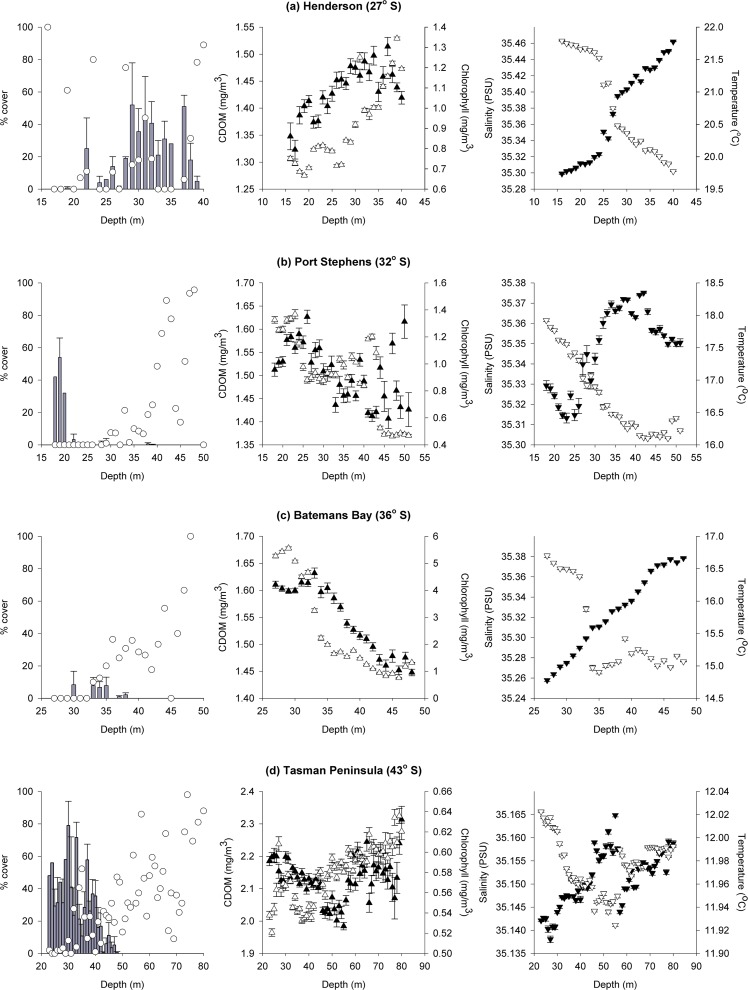
Kelp cover and physical variables across depth in East Australia. Mean (± SE) percentage cover of kelp (grey bars), sand (◯; *n* = 1–17), and *in situ* temperature (▽), salinity (▼), chlorophyll (▽) and CDOM (▼; *n* = ∼300–2000) concentration across depth at (a) Henderson (27° S), (b) Port Stephens (32° S), (c) Batemans Bay (36° S) and (d) the Tasman Peninsula (43° S).

On the East coast, kelp cover generally decreased with increasing depth and was greater in the south (Tasmania) than further north, at Batemans or Port Stephens (Fig. 5B-D). In the latter regions, kelp distribution was patchy and kelp cover was generally low (0–15%), particularly deeper than ∼30 m. Some urchin barrens (urchin density: 0.4 ± SE 0.1 m^2^) were observed between 20–30 m depths along one transect at Batemans, but these were patchy.

In Port Stephens, the combination of depth, sand, *in situ* chlorophyll-*a*, salinity and temperature was found to be the best model, explaining 76% of the variation in kelp cover (Table 3). Temperature, CDOM and chlorophyll-*a* decreased with increasing depth. The opposite was found for sand cover and salinity (Table E in S1 Appendix, Fig. 5B-D). In Batemans, the combination of CDOM, temperature and chlorophyll-*a* was found to be the best model, but explained only 38% of the variation in kelp cover (Table 3). Relationships between physical variables and depth were similar to those in Port Stephens (Table E in S1 Appendix, Table 3, Fig. 5B-D). In Tasmania, the combination of depth, temperature and chlorophyll-*a* was found to be the best model, explaining 73% of the variation in kelp cover (Table 3). Sand, chlorophyll-*a* and salinity increased with increasing depth, but no significant relationships were found between depth and temperature or CDOM (Table E in S1 Appendix, Fig. 5B-D).

**Table 3:** Regression analyses between kelp cover and physical variables at the transects in Port Stephens (PS), Batemans (Ba) and Tasman Peninsula (Ta) on East Australia.#

PS	Variable	SS(trace)	pseudo-*F*_1,30_	*P*	R^2^
Marginal tests	Depth	1360.10	10.77	**<0.01**	0.26
	Sand	293.53	1.81	0.11	0.06
	CDOM	49.23	0.29	0.60	0.01
	Chlorophyll	799.91	5.52	**0.02**	0.16
	Salinity	623.53	4.13	**0.04**	0.12
	Temperature	1816.20	16.35	**<0.01**	0.35
Sequential test	All combined				0.77
Best		BIC	R^2^		
	All except CDOM	137.95	0.76		
**Ba**	Variable	SS(trace)	pseudo-*F*_1,20_	*P*	R^2^
Marginal tests	Depth	22.18	2.64	0.13	0.12
	Sand	12.80	1.44	0.25	0.07
	CDOM	44.89	6.18	**0.02**	0.24
	Temperature	0.09	0.01	0.92	0.00
Sequential test	All combined				0.39
Best		BIC	R^2^		
	CDOM, Temperature, Chlorophyll	46.16	0.38		
**Ta**	Variable	SS(trace)	pseudo-*F*_1,53_	*P*	R^2^
Marginal tests	Depth	14561.00	77.01	**<0.01**	0.59
	Sand	9675.90	34.40	**<0.01**	0.39
	CDOM	2019.90	4.75	**0.03**	0.08
	Chlorophyll	10185.00	37.50	**<0.01**	0.41
	Salinity	11696.00	48.10	**<0.01**	0.48
	Temperature	5375.80	14.84	**<0.01**	0.22
Sequential test	All combined				0.74
Best		BIC	R^2^		
	Depth, Temperature, Chlorophyll	279.64	0.73		

^#^ The proportion of the variability explained by all variables combined was obtained in sequential tests using R^2^ selection criterion and Forward selection procedure. The overall best solution was obtained using the Bayesian Information Criterion (BIC), using all possible combinations of variables. *P*-values were calculated using 9,999 permutations. Ba: salinity and chlorophyll were removed from the model as they were strongly correlated with depth and temperature, respectively (|r|>0.95; see Table A in S1 Appendix).

### Approximate northern limit of kelp distribution on the East coast

At Henderson (27° S), kelp on reefs targeted for its presence was very abundant, with an overall mean cover of 45% ± SE 2 at a depth of ∼30 m. This was consistent between the two locations surveyed and between sites, despite significant variability among grids ∼50–100 m apart (Table 4, Fig. 3). Physical variables measured *in situ* also generally varied at the smallest spatial scale (Table F in S1 Appendix).

**Table 4:** ANOVA of kelp cover at 30 m depth at Henderson (27o S), in the northern limit of kelp distribution on the East coast of Australia.^#^

Source	*df*	MS	*F*	*P*
Location	1	90863	3	0.232
Site(Lo)	2	31624	2	0.224
Grids(Si(Lo))	8	17402	36	**<0.001**
Residual	1188	483		

^#^Location is random with 2 levels, Site is random, nested in Location, with 2 levels and Grids is random, nested in Site, with 3 levels. The replicates are the images (*n* = 100). *C =* 0.16, *P* < 0.01.

*In situ* measurements of temperature and CDOM were lower than those in the summer-winter range obtained through remote sensing. The opposite was found for chlorophyll-*a* (Fig. 3). Sea-surface temperature and PAR in Henderson were significantly higher than in the other three regions surveyed on the East coast, both in summer and winter, with the exception of PAR in Port Stephens in summer. Remote-sensed CDOM was generally lower than in the other regions, and was lowest in summer, while chlorophyll-*a* was only lower than in other regions in winter (Table C in S1 Appendix, Fig. 3). These differences in CDOM and chlorophyll-*a* varied, however, across years (Table C in S1 Appendix).

No kelp was observed shallower than 15–20 m at any of the two Henderson locations surveyed, where corals and turfing algae dominated hard substrata and *in situ* temperature was ∼22°C. Below ∼20 m, kelp cover increased with depth until ∼35 m, where the substratum increasingly became sand and kelp cover decreased (Fig. 5A). Temperature decreased with increasing depth and the opposite pattern was found for CDOM, chlorophyll-*a* and salinity. The *in situ* temperature at 30 m was lower than the mean summer sea-surface temperature for all other sites except Tasmania, while *in situ* chlorophyll-*a* was higher than at any other location. No significant relationship was found between depth and sand cover (Table G in S1 Appendix, Fig. 5A). The combination of sand and CDOM was found to be the best model, but only explained 38% of the variation in kelp cover (Table 5).

**Table 5:** Regression analyses between kelp cover and physical variables measured *in situ* at transects in Henderson, East Australia.^#^

	Variable	SS(trace)	pseudo-*F*_1,22_	*P*	R^2^
Marginal tests	Sand	1243.50	3.61	0.07	0.14
	CDOM	2292.20	7.72	**0.01**	0.26
	Chlorophyll	698.65	1.89	0.18	0.08
	Temperature	195.28	0.50	0.48	0.02
Sequential test	All combined				0.50
Best		BIC	R^2^		
	Sand, CDOM	139.83	0.38		

^#^ The proportion of the variability explained by all variables combined was obtained in sequential tests using R^2^ selection criterion and Forward selection procedure. The overall best solution was obtained using the Bayesian Information Criterion (BIC), using all possible combinations of variables. *P*-values were calculated using 9,999 permutations. Variables salinity and depth were excluded from the model as these were strongly correlated with temperature (|r|>0.95).

## Discussion

To our knowledge, this study is the first to quantify latitudinal variation in kelp distribution and abundance in deeper waters (>15 m—<80 m) at a continental scale. The total amount of the seabed surveyed (∼157,000 m^2^) was orders of magnitude higher than most other large-scale comparisons in Australian subtidal temperate reefs (*e.g*. ∼500–18,000 m^2^ [18, 40–42], but see [43] for surveys of eastern Tasmanian reefs) and other seminal long-term, large-scale studies of kelp forests elsewhere (*e.g*. ∼6,000–8,000 m^2^ [16, 44]). Kelp abundance increased significantly with increasing latitude along both coasts, with reefs in the southern-most regions having a kelp coverage of ∼50% relative to 0–30% in the more northerly sites. Kelp was generally more abundant on the west coast than on the east coast, a pattern that resembles that of shallow-water kelp forests [18]. Surprisingly, reefs at Henderson in the northern limit of kelp distribution on the east coast targeted specifically for the presence of kelp had abundances similar to reefs in the southern-most regions. Although kelp occurred at depths ∼40–50 m in all regions, the pattern of kelp abundance along the depth gradient varied across regions, with Tasmania and mid- and south WA showing the greatest covers across the depth-ranges surveyed. In central regions along both coasts, kelp coverage was greater at depths between 15–30 m, while reefs in the northern limit of kelp distribution in the east coast showed a skewed distribution towards deeper waters. These patterns establish the baseline against which future changes due to climate and other stressors could be evaluated, a necessary first step towards identifying the mechanisms to successfully manage and monitor these valuable habitats. In addition, this study constitutes a significant step towards assessing the practicality and performance of an AUV observation program in quantifying benthic habitat-forming primary producers and providing necessary information for management of conservation reserves.

The overall trend of greater kelp abundance at higher latitudes was generally related to water temperature, which is one of the most important factors influencing kelps distribution [6]. An upper thermal threshold of 18.5°C has been reported, above which *Ecklonia* adults stress, affecting their growth [33], as well as another threshold of 22°C above which the development of recruits is significantly impaired [45]. The gradual rise in ocean temperature and the increased frequency and intensity of extreme events (“heat-waves”) due to climatic change is, therefore, likely to intensify the observed gradient in kelp abundance in both coasts, and could result in the range contraction of kelp distribution [24, 26, 30, 46]. For instance, *in situ* water temperatures at Abrolhos, the northern region on the West coast, were higher than 22°C at the time of sampling (early autumn). Kelp individuals are thus likely to be physiologically stressed and further increases in water temperature predicted from climate models could limit recruitment, potentially leading to the disappearance of kelp in these areas. *In situ* temperatures were below this threshold in all regions on the East coast, but sampling was done in late spring, when water is much cooler. These temperature thresholds may, however, be population-specific and further understanding on how generalizable these thresholds are across the continent is needed. In addition, changes in temperature are likely to be more spatially variable on the east coast as cooler temperatures occur on the shelf due to wind and current-driven upwelling, the frequency, timing and duration of which vary with latitude [47] and may be altered by climatic change. High kelp coverage at Henderson, the northernmost region surveyed, is likely to be related to such processes rather than more general latitudinal trends. These data also suggest that kelp distribution at similar depths and/or in deeper waters may extend further north (depending on light and nutrient availability) and more sampling in northern latitudes may reveal the location of the northern distribution limit of this species.

Because of the stratification of the coastal water-column, the distribution of kelp may, alternatively, shift into deeper waters, particularly in northern areas where water temperature is already above or close to the thermal thresholds reported elsewhere and discussed above. This will depend, however, on the availability of resources that are essential for the establishment and survival of kelp, such as hard substrata, light and nutrients [6, 48, 49]. In this study, most of the variability in kelp cover across depth was best explained by combinations of variables such as sand cover (i.e. substratum availability), chlorophyll-*a* and/or CDOM (proxies for light and nutrient availability). In most regions surveyed in this study, hard substrata decreased with increasing depth and the seabed became increasingly dominated by sand, particularly at depths greater than 40–50 m. This restricts the possibility of deep-water refugia because kelp need hard substrata to establish new populations, and may thus result in a depth range contraction instead of a shift from shallow to deeper waters. This is analogous to the range contraction of species’ distribution on temperate coastlines in the southern hemisphere due to the limited amount of habitat available at higher latitudes, limiting poleward range shifts [50].

Light availability is likely to differ across regions, especially on the East coast where PAR values were lower at higher latitude. Light availability can also be influenced indirectly by oceanographic processes because increasing nutrients can lead to increases in phytoplankton [51], thereby reducing the light-levels reaching the seabed. Similarly, nutrient availability in deeper waters is influenced by oceanographic processes likely to vary between coasts, across regions and temporally [51, 52]. In the northern-most regions in both coasts, decreases in kelp cover across depth were strongly related to increases in chlorophyll-*a*, which may reduce light availability in deeper waters. A similar relationship was observed in Tasmania, suggesting that light availability may be influencing covers of kelp in deeper waters in these regions, in addition to substratum availability. The opposite relationship was found in Port Stephens and Batemans, where decreases in kelp cover were related to decreases in chlorophyll-*a* and CDOM, which are also proxies for nutrients, suggesting that nutrient availability may be low in deeper waters in these regions. Studies on Californian kelp forests have shown strong influences of the behaviour of thermoclines and breaking internal waves, as well as other larger scale ocean basin processes, with regards to the delivery of nutrients, which may be of greater importance than water temperature *per se* [53–55].

Relationships between kelp covers and physical variables in this study, however, need to be interpreted with caution as *in situ* measurements of physical variables have not been replicated in time and are likely to vary significantly at multiple temporal scales. Long-term observations from these regions as part of the ongoing AUV program will generate a more comprehensive dataset that will allow robust examinations and better interpretation of relationships between kelp cover and potential explanatory variables. Currently, there is little information on any of these key resources in near-shore environments, particularly inside the 50 m isobath; however, recent analysis has identified spatio-temporal patterns of variability [47]. Although fluorescence measures of CDOM and chlorophyll-*a* provide some insights of *in situ* differences in nutrient and light availability, these need to be calibrated by direct *in situ* sampling of dissolved organic carbon and chlorophyll-*a*. Mapping the seabed and quantifying spatial and temporal variability in nutrients and light will help to identify which of these resources are limiting and thus predict future changes in kelp distribution [26].

Despite similar abundances of kelp in the southern-most regions in both coasts, kelp abundance in other regions was generally lower on the east coast, which is consistent with previous observations in shallow waters [18, 27]. The main broad-scale difference between the two coasts is that they are influenced by different boundary currents. The Leeuwin Current and the Eastern Australian Current (EAC) have marked differences in flow structure, the latter being a stronger current delivering warm and nutrient-poor water to areas of the shelf in the main current, or *via* eddies, particularly at greater latitudes [26, 51]. Cooler waters, however, generally dominate near-shore waters west of the EAC [47] due to the presence of counter-currents flowing northwards [56] and/or colder water intrusions, including upwelling [57]. These and other differences between the two currents may result in differences in growth and biomass of kelp. Observed differences may also be due to the timing of the surveys. Reefs on the west coast were sampled in early autumn, while those on the east coast were sampled in late spring, with the exception of Tasmania, where sampling was done in late autumn/early winter when kelp cover is at its annual minimum. Kelp growth and biomass peak in spring/early summer and this seems to be consistent across the continent [32–34]. Because the observed differences were in the opposite direction of the growth and biomass peak (*i.e*. kelp cover in NSW on the East coast was smaller even though sampling was done during the biomass peak), it is unlikely that these differences among regions are due to differences in the time we sampled.

Kelp distribution and abundance are also influenced by ecological processes, particularly at smaller scales. Most of the variability in kelp cover within each region occurred at the spatial scales of 10’s to 100’s of metres rather than kilometres. The ecological processes influencing this pattern are likely to vary between coasts and from one region to another [27]. For instance, herbivory, particularly grazing by the sea-urchin *Centrostephanus*, is a major process influencing kelp forests on the East coast in NSW [58, 59] and Tasmania [26, 28]. In contrast, herbivory does not seem to affect kelp forests on the West coast because of functional differences in the herbivore species that occur on this coast and differences in behaviour (*e.g*. drift-feeding urchins on the west coast *vs* scraping urchins on the east coast; [60]). Physical disturbances and nutrient availability seem to be the main processes influencing small-scale variability in shallow-water western kelp forests [61], although herbivory may increase in the future as the distribution of tropical herbivores starts shifting south due to increases in water temperature [31]. The experimental work that examined these processes was, however, undertaken on shallow reefs (<15 m). In deeper waters, the effects of extreme wave action will be diminished and nutrient availability may also vary markedly with depth, therefore processes driving small scale variability in kelp cover at depth require closer examination. Further understanding of the ecological processes that drive the small-scale variability in each region will help managers develop successful conservation strategies that may ameliorate the impacts of climatic changes [24]. For example, in areas where herbivory is important and the system is driven by “top-down” processes, the implementation of no-take zones, or altered fishing regulations with the objective of increasing abundances of predators, which in turn would lead to a decrease in kelp consumers, may help to mitigate impacts due to climatic changes [26]. This, however, may be too simplistic and several interacting, local processes/stressors may need to be addressed simultaneously and at multiple scales. Some of these management actions aimed at protecting kelp habitats in both coasts are ongoing. For example, considerable advances have been made in NSW to protect *Ecklonia* dominated reefs through five large Marine Protected Areas between 28°S and 36°S that have replicated no-take zones, which will allow assessing their effects and the timeframes for potential changes following protection.

Australia’s temperate reefs support unique endemic and extremely diverse communities and valuable commercial and recreational fisheries and ecosystem services with an estimated economic value of $175B [62]. Kelp forests that dominate these reefs and provide crucial resources that sustain much of the endemic biodiversity and underpin ecosystem functioning are, however, declining due to multiple stressors such as overfishing, nutrient loading and climatic change [24, 26, 28, 46, 63], which act at different spatio-temporal scales and are likely to interact in complex ways [24, 64]. Consequently, these habitats are becoming less productive and generally support lower diversity [26, 28, 63]. Understanding the mechanisms responsible for the demise of canopy-forming seaweeds and the processes that influence their distribution and abundance is therefore necessary to develop successful strategies for conservation and management of these important ecosystems [63]. Given the variety of scales at which different stressors and processes act, this requires the combination of manipulative experiments and long-term, large-scale surveys such as those in the IMOS AUV program, specifically designed to examine predictions about the interactive effects of climatic changes and concurrent stressors.

## Supporting Information

S1 Appendix Survey locations, analyses of physical variables measured *in situ* or obtained from MODIS-Aqua 4 km (NASA) sea-surface monthly averages across all regions, and relationships between physical variables and kelp cover. (DOCX)
