# Supplementary material for: Large-Scale Geographic Variation in Distribution and Abundance of Australian Deep-Water Kelp Forests
Source: PLoS One. 2015 Feb 18;10(2):e0118390. doi: 10.1371/journal.pone.0118390 (PMC4334971; doi:10.1371/journal.pone.0118390)
Supplement: S1 Appendix — (DOCX) [file pone.0118390.s001.docx]

**Supporting Information**

Survey locations, analyses of physical variables measured *in situ* or obtained from MODIS-Aqua 4 km (NASA) sea-surface monthly averages across all regions, and relationships between physical variables and kelp cover.

**Table A.** Coordinates of (a) 25 x 25 m grids and (b) 200-500 x 1.5 m transects surveyed across Australia in 2010 with >60% cover of reef habitat.

|  | **Coast** | **Region** | **Depth (m)** | **Location** | **Site** | **Grid** | **Latitude (S)** | **Longitude (E)** |
| --- | --- | --- | --- | --- | --- | --- | --- | --- |
| **(a) Grids** | West | Abrolhos | 15 | Ab-1 |  | 1 | 28.8485 | 114.0278 |
|  | West | Abrolhos | 15 | Ab-1 |  | 2 | 28.8476 | 114.0284 |
|  | West | Abrolhos | 15 | Ab-1 |  | 3 | 28.8467 | 114.0289 |
|  | West | Abrolhos | 30 | Ab-1 |  | 4 | 28.8588 | 114.0313 |
|  | West | Abrolhos | 30 | Ab-1 |  | 5 | 28.8585 | 114.0321 |
|  | West | Abrolhos | 30 | Ab-1 |  | 6 | 28.8576 | 114.0326 |
|  | West | Abrolhos | 30 | Ab-2 |  | 7 | 28.8073 | 113.9612 |
|  | West | Abrolhos | 40 | Ab-1 |  | 8 | 28.8471 | 114.0470 |
|  | West | Abrolhos | 40 | Ab-1 |  | 9 | 28.8471 | 114.0441 |
|  | West | Jurien | 30 | Ju-1 |  | 10 | 30.3760 | 114.9764 |
|  | West | Jurien | 30 | Ju-1 |  | 11 | 30.3757 | 114.9746 |
|  | West | Jurien | 30 | Ju-1 |  | 12 | 30.3753 | 114.9752 |
|  | West | Jurien | 40 | Ju-1 |  | 13 | 30.3268 | 114.7859 |
|  | West | Jurien | 40 | Ju-1 |  | 14 | 30.3262 | 114.7849 |
|  | West | Jurien | 40 | Ju-1 |  | 15 | 30.3256 | 114.7839 |
|  | West | Rottnest | 15 | Ro-1 |  | 16 | 32.0121 | 115.4463 |
|  | West | Rottnest | 15 | Ro-1 |  | 17 | 32.0117 | 115.4462 |
|  | West | Rottnest | 15 | Ro-1 |  | 18 | 32.0128 | 115.4458 |
|  | West | Rottnest | 30 | Ro-1 |  | 19 | 32.0205 | 115.4410 |
|  | West | Rottnest | 30 | Ro-1 |  | 20 | 32.0202 | 115.4402 |
|  | West | Rottnest | 30 | Ro-1 |  | 21 | 32.0210 | 115.4402 |
|  | West | Rottnest | 30 | Ro-2 |  | 22 | 32.0347 | 115.4602 |
|  | West | Rottnest | 30 | Ro-2 |  | 23 | 32.0343 | 115.4599 |
|  | West | Rottnest | 30 | Ro-2 |  | 24 | 32.0339 | 115.4596 |
|  | West | Rottnest | 40 | Ro-1 |  | 25 | 32.0147 | 115.4208 |
|  | West | Rottnest | 40 | Ro-1 |  | 26 | 32.0140 | 115.4210 |
|  | West | Rottnest | 40 | Ro-1 |  | 27 | 32.0142 | 115.4207 |
|  | East | Henderson | 30 | HN | 1 | 34 | 27.0764 | 153.4772 |
|  | East | Henderson | 30 | HN | 1 | 35 | 27.0758 | 153.4772 |
|  | East | Henderson | 30 | HN | 1 | 36 | 27.0752 | 153.4772 |
|  | East | Henderson | 30 | HN | 2 | 37 | 27.0746 | 153.4773 |
|  | East | Henderson | 30 | HN | 2 | 38 | 27.0744 | 153.4773 |
|  | East | Henderson | 30 | HN | 2 | 39 | 27.0741 | 153.4773 |
|  | East | Henderson | 30 | HS | 3 | 40 | 27.1250 | 153.4754 |
|  | East | Henderson | 30 | HS | 3 | 41 | 27.1251 | 153.4754 |
|  | East | Henderson | 30 | HS | 3 | 42 | 27.1254 | 153.4754 |
|  | East | Henderson | 30 | HS | 4 | 43 | 27.1241 | 153.4754 |
|  | East | Henderson | 30 | HS | 4 | 44 | 27.1237 | 153.4754 |
|  | East | Henderson | 30 | HS | 4 | 45 | 27.1239 | 153.4754 |
|  | East | Port Stephens | 30 | PS-1 |  | 46 | 32.7537 | 152.1975 |
|  | East | Port Stephens | 30 | PS-1 |  | 47 | 32.7512 | 152.1976 |
|  | East | Port Stephens | 30 | PS-1 |  | 48 | 32.7538 | 152.1976 |
|  | East | Port Stephens | 30 | PS-2 |  | 49 | 32.6340 | 152.3101 |
|  | East | Port Stephens | 30 | PS-2 |  | 50 | 32.6318 | 152.3101 |
|  | East | Port Stephens | 30 | PS-2 |  | 51 | 32.6311 | 152.3101 |
|  | East | Batemans Bay | 30 | Ba-1 |  | 28 | 35.7585 | 150.2754 |
|  | East | Batemans Bay | 30 | Ba-1 |  | 29 | 35.7567 | 150.2755 |
|  | East | Batemans Bay | 30 | Ba-1 |  | 30 | 35.7523 | 150.2736 |
|  | East | Batemans Bay | 30 | Ba-2 |  | 31 | 35.9643 | 150.0946 |
|  | East | Batemans Bay | 30 | Ba-2 |  | 32 | 35.9641 | 150.1751 |
|  | East | Batemans Bay | 30 | Ba-2 |  | 33 | 35.9650 | 150.1797 |
|  | East | Tasmania | 30 | Ta-1 |  | 52 | 43.1475 | 148.0046 |
|  | East | Tasmania | 30 | Ta-1 |  | 53 | 43.1475 | 148.0046 |
|  | East | Tasmania | 30 | Ta-2 |  | 54 | 41.8675 | 148.3013 |
|  | East | Tasmania | 30 | Ta-2 |  | 55 | 41.8647 | 148.3002 |
|  | East | Tasmania | 30 | Ta-3 |  | 56 | 43.5456 | 147.3012 |
|  | East | Tasmania | 30 | Ta-3 |  | 57 | 43.5253 | 147.2998 |
|  | East | Tasmania | 30 | Ta-4 |  | 58 | 42.6406 | 148.1551 |
|  | East | Tasmania | 30 | Ta-4 |  | 59 | 42.6406 | 148.1551 |
|  | East | Tasmania | 30 | Ta-5 |  | 60 | 42.1141 | 148.3575 |
|  | East | Tasmania | 30 | Ta-5 |  | 61 | 42.1194 | 148.3582 |
| **(b) Transects** | East | Henderson |  | HN | 1 |  | 27.0815 | 153.4769 |
|  | East | Henderson |  | HS | 1 |  | 27.1301 | 153.4751 |
|  | East | Port Stephens |  | PS-1 | 1 |  | 32.7538 | 152.1973 |
|  | East | Port Stephens |  | PS-2 | 1 |  | 32.6246 | 152.3395 |
|  | East | Batemans Bay |  | Ba-1 | 1 |  | 35.7557 | 150.2751 |
|  | East | Batemans Bay |  | Ba-1 | 2 |  | 35.7506 | 150.2799 |
|  | East | Tasmania |  | Ta-1 | 1 |  | 43.1210 | 148.0388 |
|  | East | Tasmania |  | Ta-1 | 2 |  | 43.1197 | 148.0466 |

**Table B.** ANOVA of *in situ* temperature (^o^C), salinity (PSU), chlorophyll and CDOM concentration (mg/m^3^) at 30 m depth among regions separated by 2^o^ to 4^o^ latitude in (a) Western Australia and (b) New South Wales, and (c) among locations in Tasmania.^#^

|  |  | **Temperature** | | **Salinity** | | **Chlorophyll** | | **CDOM** | |
| --- | --- | --- | --- | --- | --- | --- | --- | --- | --- |
| **(a)** Source | *df* | MS | *F* | MS | *F* | MS | *F* | MS | *F* |
| Region | 2 | 278.512 | 414** | 10.6273 | 84** | 27.609 | 150** | 11.3 | 8.1* |
| Grids(Re) | 6 | 0.672 | 133** | 0.1267 | 610** | 0.185 | 47** | 1.4 | 6.8** |
| Residual | 891 | 0.005 |  | 0.0002 |  | 0.004 |  | 0.2 |  |
|  | *C*:  Contrasts: | 0.96**  Ab > Ro > Ju | | 0.52**  Ro = Ju > Ab | | 0.35**  Ab > Ro > Ju | | 0.52**  Ju > Ab = Ro | |
| **(b)** Source | *df* | MS | *F* | MS | *F* | MS | *F* | MS | *F* |
| Region | 1 | 871.94 | 0.7 ns | 0.1578 | 0.2 ns | 24.69 | 0.3 ns | 5.7 | 0.6 ns |
| Location(Re) | 2 | 1176.11 | 57.7** | 0.9546 | 35.3** | 89.06 | 42.6** | 9.2 | 49.3** |
| Grids(Lo(Re)) | 8 | 20.40 | 976.5** | 0.0270 | 61.4** | 2.09 | 40.2** | 0.2 | 2.4* |
| Residual | 1188 | 0.02 |  | 0.0004 |  | 0.05 |  |  |  |
|  | *C*: | 0.28** | | 0.24** | | 0.26** | | 0.13* | |
| **(c)** Source | *df* | MS | *F* | MS | *F* | MS | *F* | MS | *F* |
| Location | 4 | 11.9270 | 2 ns | 0.8351 | 4 ns | 1.242 | 2 ns | 5.6 | 2 ns |
| Grids(Lo) | 5 | 6.0349 | 107763** | 0.2341 | 18494** | 0.769 | 654** | 3.0 | 40** |
| Residual | 190 | 0.0001 |  | 0.0000 |  | 0.001 |  | 0.1 |  |
|  | *C*: | 0.29** | | 0.19 ns | | 0.22** | | 0.22* | |

^#^ Cochran’s test (*C*) was used to test assumption of homogeneous variances. Transformation of data failed to make variances homogeneous; analyses were still done using untransformed data. ns, *P* > 0.05; *, *P* < 0.05; **, *P* < 0.01. (a) Region was fixed with 3 levels (Ab, Abrolhos 28^o^ S; Ju, Jurien 30^o^ S; Ro, Rottnest 32^o^ S), Grids was random, nested in Region, with 3 levels (*n* = 100). (b) Region was fixed with 2 levels (PS, Port Stephens 32^o^ S; Ba, Batemans 36^o^ S), Location was random, nested in Region with 2 levels, Grids was random, nested in Location, with 3 levels (*n* = 100). (c) Location was random with 5 levels (all between ~42-43^o^ S), Grids was random, nested in Location, with 2 levels (*n* = 20).

**Table C.** ANOVA of monthly averages of sea-surface temperature (^o^C), PAR (Einstein/m^2^/day), chlorophyll concentration (mg/m^3^) and CDOM index (MODIS-Aqua 4 km, NASA) among regions separated by 2^o^ to 4^o^ latitude along the (a) West and (b) East coasts of Australia.^#^

| **(a) West** |  | **Temperature** | | **PAR** | | **CDOM** | | **Chlorophyll** | |
| --- | --- | --- | --- | --- | --- | --- | --- | --- | --- |
| Source | *df* | MS | *F* | MS | *F* | MS | *F* | MS | *F* |
| Re | 2 | 29.17 | 75.71** | 125.02 | 2.76 ns | 107.37 | 46.04** | 5.12 | 1.26 ns |
| Lo(Re) | 3 | 0.03 | Pooled | 0.44 | Pooled | 2.33 | 2.22 ns | 4.07 | 160.24** |
| Se | 1 | 406.65 | 155.26** | 36709.39 | 811.03** | 7.00 | 0.47 ns | 0.38 | 14.90** |
| Yr | 7 | 4.06 | 10.53** | 19.65 | 0.43 ns | 8.37 | 7.98** | 0.02 | 0.84 ns |
| RexSe | 2 | 0.04 | 0.09 ns | 46.18 | 1.02 ns | 8.49 | 8.10** | 0.33 | 12.96** |
| RexYr | 14 | 0.03 | Pooled | 6.47 | Pooled | 0.42 | Pooled | 0.03 | Pooled |
| SexLo(Re) | 3 | 0.21 | Pooled | 0.30 | Pooled | 1.03 | Pooled | 0.22 | Pooled |
| YrxLo(Re) | 21 | 0.01 | Pooled | 0.21 | Pooled | 0.29 | Pooled | 0.02 | Pooled |
| SexYr | 7 | 2.62 | 6.80** | 29.25 | Pooled | 14.93 | 14.24** | 0.04 | Pooled |
| RexSexYr | 14 | 0.19 | Pooled | 2.93 | Pooled | 0.92 | Pooled | 0.03 | Pooled |
| YrxSexLo(Re) | 21 | 0.00 | Pooled | 0.23 | Pooled | 0.29 | Pooled | 0.03 | Pooled |
| Residual | 192 | 0.52 |  | 63.02 |  | 1.27 |  | 0.02 |  |
|  | *C*: | 0.04 ns | | 0.03 ns | | 0.07 ns | | 0.22** | |
| Contrasts: Regions:  Season: | | Ab>Ju>Ro  S>W all Yr | | S>W | | S: Ju>Ro>Ab  W: Ju>Ro=Ab  Ab,Ro: S=W  Ju: S>W  3 Yr: S<W  3 Yr: S>W  2 Yr: S=W | | S,W: Ab>Ju>Ro  Ab: S=W  Ju,Ro: S<W | |
| **(b) East** |  | **Temperature** | | **PAR** | | **CDOM** | | **Chlorophyll** | |
| Source | *df* | MS | *F* | MS | *F* | MS | *F* | MS | *F* |
| Re | 2 | 18.35 | No test | 2126.72 | No test | 1.99 | No test | 10.23 | No test |
| Lo(Re) | 3 | 0.01 | 2.71 * | 4.74 | 0.10 ns | 0.22 | 2.75 * | 0.21 | 0.25 ns |
| Se | 1 | 28.71 | No test | 29959.83 | 2668.86** | 0.13 | 0.18 ns | 11.80 | 11.93 * |
| Yr | 7 | 0.06 | 15.94** | 7.83 | 0.17 ns | 0.24 | 2.94** | 1.43 | 1.77 ns |
| RexSe | 2 | 0.10 | No test | 206.84 | 4.48** | 0.97 | 11.97** | 7.38 | 6.33** |
| RexYr | 14 | 0.02 | 6.91** | 26.93 | 0.58 ns | 0.17 | 2.12** | 1.39 | 1.73 * |
| SexLo(Re) | 3 | 0.00 | 0.11 ns | 2.18 | Pooled | 0.09 | Pooled | 0.27 | Pooled |
| YrxLo(Re) | 21 | 0.00 | Pooled | 0.45 | Pooled | 0.05 | Pooled | 0.62 | Pooled |
| SexYr | 7 | 0.01 | 3.09** | 11.23 | 0.24 ns | 0.72 | 8.95** | 0.99 | 1.22 ns |
| RexSexYr | 14 | 0.01 | 3.00** | 13.71 | Pooled | 0.10 | Pooled | 1.17 | 1.44 ns |
| YrxSexLo(Re) | 21 | 0.00 | Pooled | 0.62 | Pooled | 0.05 | Pooled | 0.54 | Pooled |
| Residual | 192 | 0.00 |  | 59.54 |  | 0.09 |  | 0.87 |  |
|  | *C*: | 0.04 ns | | 0.03 ns | | 0.07 ns | | 0.22** | |
| Contrasts: Regions:  Season: | | He>PS>Ba>Ta  all Yr & Se  S>W all Yr & Re | | S: He=PS>Ba=Ta  W: He>PS=Ba>Ta  S>W all Yr & Re | | S:He<PS=Ba=Ta  W:He=Ba<PS=Ta  4 Yr: He=PS=Ba=Ta  1 Yr: He=PS=Ba<Ta  3 Yr: He<PS=Ba=Ta  PS,Ta: S=W  Ba:S>W,He:S<W  3 Yr: S<W  2 Yr: S>W  3 Yr: S=W | | S:PS>He=Ba=Ta  W:He<PS=Ba=Ta  4 Yr: He=PS=Ba=Ta  2 Yr: PS>He=Ba=Ta  1 Yr: He<PS=Ba=Ta  1 Yr: Ba>He=PS=Ta  He,PS,Ta: S=W  Ba: S<W | |

^#^Cochran’s test (*C*) was used to test assumption of homogeneous variances. Non-significant terms with *P* > 0.25 were pooled. ns, *P* > 0.05; *, *P* < 0.05; **, *P* < 0.01. Data for temperature and CDOM in (b) were square-root transformed. Region was fixed, with (a) 3 (Ab, Abrolhos 28^o^ S; Ju, Jurien 30^o^ S; Ro, Rottnest 32^o^ S) or (b) 4 levels (He, Henderson 27^o^ S; PS, Port Stephens 32^o^ S; Ba, Batemans 36^o^ S; Ta, Tasmania 42^o^ S), Location was random, nested in Region, with 2 levels, Season was fixed, with 2 levels (summer S *vs* winter W), Year was random, with 8 levels. The replicates were the monthly averages (*n* = 3).

**Table D.** ANOVA of *in situ* temperature (^o^C), salinity (PSU), chlorophyll and CDOM concentration (mg/m^3^) at (a) 15, 30 and 40 m at Abrolhos and Rottnest and (b) at 30 and 40 m at Abrolhos, Jurien and Rottnest in Western Australia. ^#^

|  |  | **Temperature** | | **Salinity** | | **Chlorophyll** | | **CDOM** | |
| --- | --- | --- | --- | --- | --- | --- | --- | --- | --- |
| **(a)** Source | *df* | MS | *F* | MS | *F* | MS | *F* | MS | *F* |
| Region | 1 | 693.21 | 275** | 45.209 | 110** | 135.461 | 488** | 4.3 | 4 ns |
| Depth | 2 | 14.33 | 6 * | 0.293 | 1 ns | 37.767 | 136** | 3.8 | 3 ns |
| Re x De | 2 | 98.02 | 39** | 0.630 | 2 ns | 12.190 | 44** | 4.7 | 4 * |
| Grids(Re x De) | 12 | 2.53 | 191** | 0.411 | 357** | 0.278 | 34** | 1.1 | 12** |
| Residual | 1782 | 0.01 |  | 0.001 |  | 0.008 |  | 0.1 |  |
| *C*:  Contrasts: | | 0.54**  15m: Ab > Ro  30m: Ab > Ro  40m: Ab > Ro  Ab: 15=30 > 40  Ro: 15 < 30=40 | | 0.76**  Ro >Ab | | 0.41**  15m: Ab > Ro  30m: Ab > Ro  40m: Ab > Ro  Ab: 15 < 30 < 40  Ro: 15 < 30=40 | | 0.07 ns  15m: Ro > Ab  30,40m: Ro=Ab  Ab: 15=30=40  Ro: 15=30=40 | |
| **(b)** Source | *df* | MS | *F* | MS | *F* | MS | *F* | MS | *F* |
| Region | 2 | 168.830 | 67** | 11.815 | 29** | 95.165 | 340** | 1.2 | 1 ns |
| Depth | 1 | 44.359 | 18** | 3.910 | 10** | 6.760 | 24** | 4.1 | 3 ns |
| Re x De | 2 | 134.184 | 54** | 8.197 | 20** | 6.786 | 24** | 13.6 | 11** |
| Grids(Re x De) | 12 | 2.519 | 263** | 0.409 | 419** | 0.280 | 33** | 1.2 | 8** |
| Residual | 1782 | 0.009 |  | 0.001 |  | 0.008 |  | 0.2 |  |
| *C*:  Contrasts: | | 0.74**  30m: Ab > Ro> Ju  40m: Ab=Ju > Ro  Ab: 30 > 40  Ju: 30 < 40  Ro: 30=40 | | 0.89**  30m: Ab < Ju =Ro  40m: Ab=Ju < Ro  Ab: 30=40  Ju: 30 > 40  Ro: 30=40 | | 0.41**  30m: Ab >Ju=Ro  40m: Ab >Ju=Ro  Ab: 30 < 40  Ju: 30=40  Ro: 30=40 | | 0.36**  30m: Ju>Ab=Ro  40m: Ab=Ju=Ro  Ab: 30=40  Ju: 30 > 40  Ro: 30=40 | |

^#^Cochran’s test (*C*) was used to test assumption of homogeneous variances. Transformation of data failed to make variances homogeneous; analyses were still done using untransformed data. ns, *P* > 0.05; *, *P* < 0.05; **, *P* < 0.01. (a) Region was fixed with 2 levels (Ab, Abrolhos 28^o^ S; Ro, Rottnest 32^o^ S), Depth was fixed, orthogonal, with 3 levels (15, 30, 40 m). Grids was random, nested in Region and Depth, with 3 levels (*n* = 100). (b) Region was fixed with 3 levels (Ab, Abrolhos 28^o^ S; Ju, Jurien 30^o^ S; Ro, Rottnest 32^o^ S), Depth was fixed, orthogonal, with 2 levels (30, 40 m). Grids was random, nested in Region and Depth, with 3 levels (*n* = 100).

**Table E.** Correlations among depth, sand cover and the physical variables measured *in situ* at the transects in Port Stephens (PS), Batemans (Ba) and Tasman Peninsula (Ta) on East Australia.^#^

| **PS** (*n*=32) | Depth | Sand | CDOM | Chlorophyll | Salinity |
| --- | --- | --- | --- | --- | --- |
| Sand | **0.71** |  |  |  |  |
| CDOM | **-0.50** | **-0.56** |  |  |  |
| Chlorophyll | **-0.79** | **-0.41** | 0.15 |  |  |
| Salinity | **0.71** | **0.45** | **-0.73** | -0.34 |  |
| Temperature | **-0.93** | **-0.64** | **0.63** | **0.66** | **-0.88** |
| **Ba** (*n*=22) | Depth | Sand | CDOM | Chlorophyll | Salinity |
| Sand | **0.77** |  |  |  |  |
| CDOM | **-0.94** | **-0.71** |  |  |  |
| Chlorophyll | **-0.90** | **-0.65** | **0.78** |  |  |
| Salinity | **0.99** | **0.73** | **-0.93** | **-0.93** |  |
| Temperature | **-0.79** | **-0.62** | **0.65** | **0.96** | **-0.83** |
| **Ta** (*n*=55) | Depth | Sand | CDOM | Chlorophyll | Salinity |
| Sand | **0.72** |  |  |  |  |
| CDOM | 0.21 | -0.03 |  |  |  |
| Chlorophyll | **0.79** | **0.60** | 0.03 |  |  |
| Salinity | **0.68** | **-0.50** | **-0.49** | **0.45** |  |
| Temperature | -0.17 | -0.15 | **0.73** | 0.03 | **-0.70** |

^#^Pearson’s r values in bold: *P* < 0.05.

**Table F.** ANOVA of *in situ* temperature (^o^C), salinity (PSU), chlorophyll and CDOM concentration (mg/m^3^) at 30 m depth at two locations in Henderson (27^o^ S), the northern limit of kelp distribution on the East coast of Australia.^#^

|  |  | **Temperature** | | **Salinity** | | **Chlorophyll** | | **CDOM** | |
| --- | --- | --- | --- | --- | --- | --- | --- | --- | --- |
| **(a)** Source | *df* | MS | *F* | MS | *F* | MS | *F* | MS | *F* |
| Location | 1 | 60.50 | 0.62 ns | 0.46 | 0.38 ns | 34.79 | 13.68** | 0.06 | 0.04 ns |
| Site(Lo) | 2 | 97.89 | 2.77 ns | 1.20 | 2.84 ns | 2.54 | pooled | 1.73 | 7.36 * |
| Grids(Si(Lo)) | 8 | 35.36 | 422.15** | 0.42 | 195.12** | 3.31 | 126.65** | 0.24 | 3.26** |
| Residual | 1188 | 0.08 |  | 0.00 |  | 0.03 |  | 0.07 |  |
|  | *C*: | 0.39** | | 0.51** | | 0.19** | | 0.11 ns | |

^#^Cochran’s test (*C*) was used to test assumption of homogeneous variances. Transformation of data failed to make variances homogeneous; analyses were still done using untransformed data. Non-significant terms with *P* > 0.25 were pooled. ns, *P* > 0.05; *, *P* < 0.05; **, *P* < 0.01. Location was random, with 2 levels, Site was random, nested in Location, with 2 levels, Grids was random, nested in Site, with 3 levels (*n* = 100).

**Table G.** Correlations among depth, sand cover and the physical variables measured *in situ* at transects in Henderson, East Australia. ^#^

| *n*=24 | Depth | Sand | CDOM | Chlorophyll | Salinity |
| --- | --- | --- | --- | --- | --- |
| Sand | 0.21 |  |  |  |  |
| CDOM | **0.66** | -0.06 |  |  |  |
| Chlorophyll | **0.82** | 0.28 | **0.50** |  |  |
| Salinity | **0.92** | 0.31 | **0.49** | **0.79** |  |
| Temperature | **-0.97** | -0.25 | **-0.59** | **-0.85** | **-0.98** |

^#^ Pearson’s r values in bold: *P* < 0.05.
